# Diseases and Injuries of the Teeth, Including Pathology and Treatment

**Published:** 1894-02

**Authors:** 


### Bibliography.

Diseases and Injuries of the Teeth, including Pathology and Treatment. A Manual of Practical Dentistry for Students and Practitioners. By Morton Smale, M.R.C.S., L.S.A., L.D.S., Dental Surgeon to St. Mary's Hospital, Dean of the School, Dental Hospital of London, etc., and J. F. Colyer, L.R.C.P., M.R.C.S., L.D.S., Assistant Dental Surgeon to Charing Cross Hospital, etc. Longman, Green & Co., London and New York, 1893.

This volume of four hundred and twenty-three pages is a welcome addition to the list of books relating to dentistry. A practical work on dental pathology has been long needed. Several very valuable contributions have been published from time to time of more or less ability, but they have not met the pressing need of students for a text-book or manual covering all the essential facts and yet not omitting detailed thoroughness in description. This book by Drs. Smale and Colyer comes nearer filling this want than any yet published, but while it is prepared with marked ability on the scientific side of each question discussed, it fails to enter into details on most of the practical points. This mental twist, so to speak, has been noticed before in books issued in England, and it is this which has made it impossible to use them with any advantage on this side of the ocean.

This is less open to objection than any issued from the English press, and if before it reaches a second edition changes are made to bring it more nearly in line with the daily practical work of students, it will hold a very important place among the text-books.

The work opens with a chapter on first dentition, followed by twenty-two chapters covering nearly all the subjects embraced under what is generally understood as dental pathology.

Very curiously, the "Introduction," in connection with other subjects, takes up the question of fees. "The question of fee is always a difficult one to write about, but . . . the dental surgeon is not selling materials, whether for operative or mechanical dentistry. Whatever fee he may elect to name should be for his skill and experience."

This sentiment will, probably, be cordially endorsed everywhere.

The chapters on "Dentition," I. and III., are, as a whole, well prepared and satisfactorily illustrated, but there are some points not well described. The remark that "*Local disturbances accompanying eruption of the permanent teeth*" are mainly confined to the third molars may be passed over with the remark that facts do not sustain it, as it is not unusual to have serious reflex disturbance from the second molar, and good reasons could be given why this should be so.

The paragraphs relating to the third molar are so surprisingly defective in statement that the reviewer is astonished that the authors should have been willing to have it go out to the world that this is all they could say on this important topic. The eruption of the third molar with its possible three important malpositions should be given more than a page and a half in a book such as this. The treatment is good as far as it goes, but as a whole is not satisfactory.

Pericementitis is fairly well described, but in the treatment the conditions pre-existing in the pulp-canal are entirely ignored. That these are the cause and must precede all apical pericementitis is clearly understood, and should have been made clear in the text. It is the old story of counter-irritation and systemic treatment, leaving out the principal factor in the inflammation.

The same criticism applies to pyorrhœa alveolaris. It could hardly be expected that the treatment given should be a success, but then this is by no means peculiar to this book or to England. The complaint is universal, "We cannot cure this disease;" yet some do; why this difference?

In the devitalization of the pulp the authors follow recognized procedures, but seem to have failed to recognize the fact that hyperæmia present will prevent absorption of arsenic and produce violent pain. They present no method to meet this, in fact do not mention it as a possible contingency.

The chapter on "Caries" is most excellently prepared. The subject-matter is well stated, and the photo-micrographs beautifully exhibit the entire process. Indeed, we have rarely seen them better done.

We have not space to follow the authors throughout their work, nor have we any wish to be thought hypercritical. The desire has been that by calling attention to some defects a remedy may be applied before a second edition is presented. We can say of this book as we can of no other with which we are familiar, that with the changes suggested it would be the most satisfactory in dental pathology yet presented as a text-book.

The practice of sending a book out with the leaves uncut is simply detestable. This may be "good form" in England, but it is certainly sure not to be commended in America, where time counts for a great deal.

In other respects the publishers have spared no pains or expense in producing the work in the best manner.

